# American Gynæcological Association

**Published:** 1880-10

**Authors:** 


American Gynæcological Association.—The third annual meeting of this body at Cincinnati, is reported to have been one of unusual interest and importance in the value of many of the papers read, and the discussions that ensued thereon. President Sims' address was able, manly and suggestive, as to what the society *should* do in order to become what it ought to be and must be; otherwise, new and rival organizations may arise *and* absorb much valuable talent that would come into it under more liberal management and rules. We think we see, however, a too greatly increasing fondness among certain parties in the profession, to be known, recognized, and as belonging to the family of Gynæcological specialists. We have seen their pertness and flippancy, and all have heard that even vulgar jests, and obscene allusions and innuendo, have been perpetrated before students in connection with the subject. It would appear, that such regard this *most sacred* and *delicate* department of the profession, as about *on a par* with the blacksmith's hammer or other trade they may have followed in earlier days. It would not add to the respectability of the societies which may be formed, nor to the existing one either, to receive into or continue the membership of men who were not bred and educated in the refinement customary among gentlemen. If delicacy and gentleness with refinement, are important for a physician who is necessarily brought into contact with the inner life of society, so to speak, they are or should be *made indispensable pre-requisites* to admission into the sacred precincts of the most important and delicate *of all* the positions which a medical man *can* sustain to the suffering portion of the female sex, when he assumes the roll of gynæcologist in *any* community, and especially so, when he is brought into professional contact with refined and educated ladies! The originator of the specialty, and his co-adjutors and confreres, are *all gentlemen of this stamp*, and unless a man shall be found to measure fully up to the standard of our Sims', Thomas' Taylor's Barker's, Emmett's, Goodall's, Chadwick's, Campbell's, *et genus omne ; he should in no wise* be permitted to become a member of a gynæcological society, and if possible, restrained from practicing *that* department, if indeed *he should* become a physician at all. After electing the following officers for the ensuing year, the society adjourned, to meet in New York City, on the 3rd Wednesday in September, 1881, viz:

President, Dr. W. H. Byford, of Chicago; Vice President, Dr. T. A. Reamy, of Cincinnati; Dr. H. F. Campbell, of Augusta, Ga., Secretary, Dr. P. F. Munde, of New York, Council; Dr. A. H. Smith, of Philadelphia, Dr. J. C. Reaves, of Dayton, Ohio, Dr. J. D. Lyman, Boston, and Dr. J. T. Johnson, of Washington, D. C.

DR. J. MARION SIMS, of New York, had the decoration of the order of Leopold I, tendered him seventeen years ago by the King of Belgium, but was prevented from receiving it through the unwanted interposition of the then American minister, whose prejudice against him, because of his sympathy for the southern people, among whom he was born and reared, was greater than his wisdom, has recently received the decoration.

We congratulate our worthy countryman upon his final success, and hope he may live long to wear this and other honors he has so richly earned.

